# Supplementary material for: Scouring the human Hsp70 network uncovers diverse chaperone safeguards buffering TDP-43 toxicity
Source: bioRxiv. 2025 May 10:2025.05.10.653282. Preprint. [Version 1] doi: 10.1101/2025.05.10.653282 (PMC12248113; doi:10.1101/2025.05.10.653282)
Supplement: 1 [file NIHPP2025.05.10.653282V1-supplement-1.pdf]

bioRxiv preprint doi: https://doi.org/10.1101/2025.05.10.653282; this version posted May 10, 2025. The copyright holder for this preprint (which was not certified by peer review) is the author/funder, who has granted bioRxiv a license to display the preprint in perpetuity. It is made available under aCC-BY 4.0 International license.

Table S1. Ranked summary of Hsp70 network components screened for suppression of TDP-43 toxicity in yeast. Proteins are listed in order of decreasing growth rescue (TDP-43 Mean Growth/Vector). Chaperones with a statistically significant difference from vector control ($p < 0.05$) determined by one-way ANOVA and Dunnett's multiple comparisons test. The table includes each protein's UniProt-reported subcellular localization, and its compartment association based on experimental data[30].

bioRxiv preprint doi: https://doi.org/10.1101/2025.05.10.653282; this version posted May 10, 2025. The copyright holder for this preprint (which was not certified by peer review) is the author/funder, who has granted bioRxiv a license to display the preprint in perpetuity. It is made available under aCC-BY 4.0 International license.

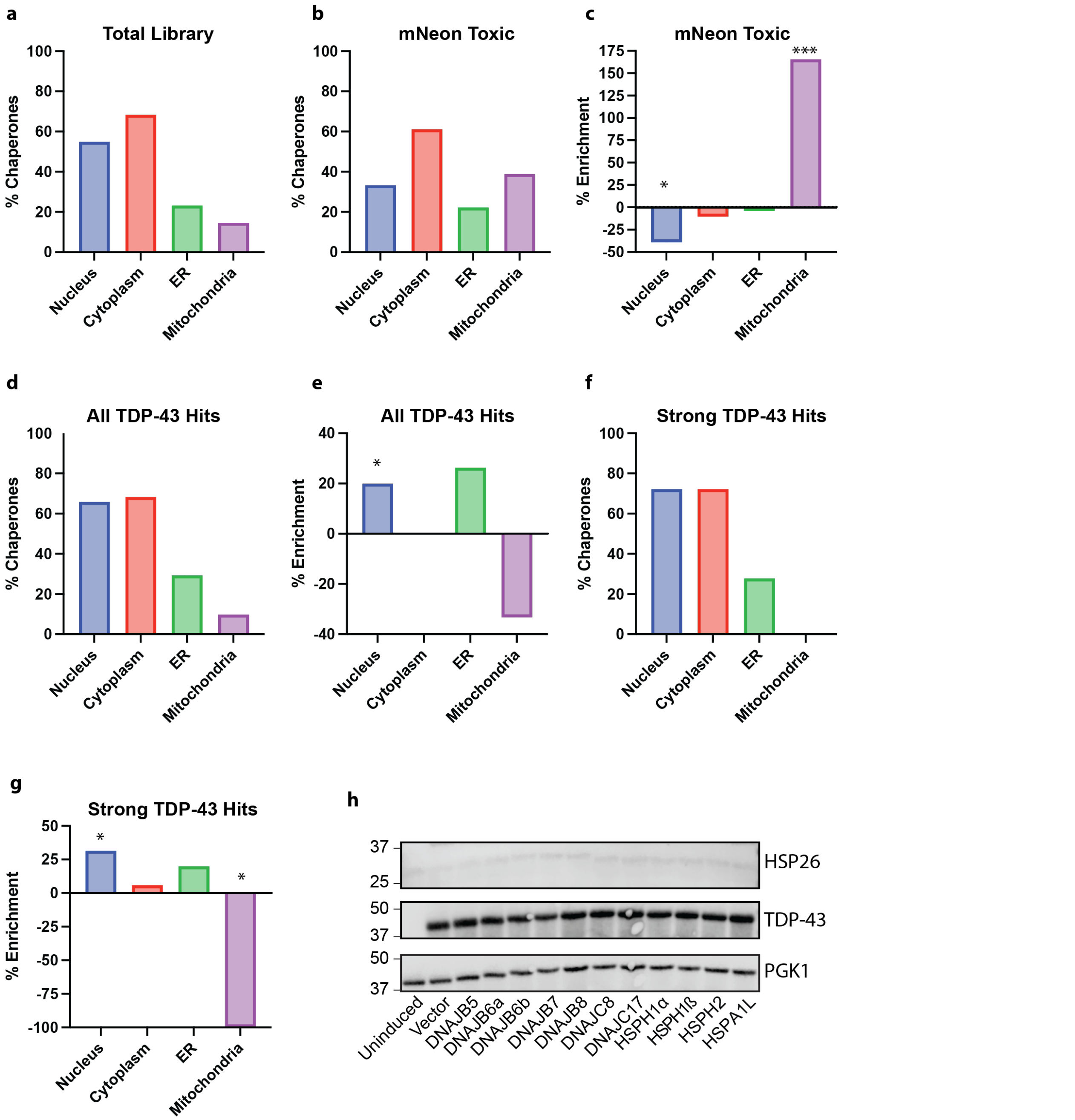

Supplementary Figure 1. Components of the human Hsp70 network that mitigate TDP-43 toxicity localize to the nucleus, ER, and cytoplasm and do not induce a general stress response. (A) Distribution of subcellular localization among all human Hsp70 network components included in the screen, categorized as nuclear, cytoplasmic, ER, or mitochondrial (%). The total exceeds 100% because many chaperones are associated with multiple subcellular compartments (see Table S1). **(B)** Distribution of subcellular localization for human Hsp70 network components that reduced growth by >10% in the mNeon-expressing control strain (%). **(C)** Enrichment or depletion (%) of human Hsp70 network components from each subcellular compartments that impaired growth in the mNeon control strain relative to the total library. **(D)** Distribution of subcellular localization for the 41 human Hsp70 network components that enhanced growth in the TDP-43-expressing strain (%). **(E)** Enrichment or depletion (%) of the 41

bioRxiv preprint doi: https://doi.org/10.1101/2025.05.10.653282; this version posted May 10, 2025. The copyright holder for this preprint (which was not certified by peer review) is the author/funder, who has granted bioRxiv a license to display the preprint in perpetuity. It is made available under aCC-BY 4.0 International license.

human Hsp70 network components from each subcellular compartment that enhanced growth in the TDP-43 strain relative to the total library. **(F)** Distribution of subcellular localization for human Hsp70 network components that strongly enhanced growth (>50%) in the TDP-43-expressing strain (%). **(G)** Enrichment or depletion (%) of human Hsp70 network components from each subcellular compartment that strongly enhanced growth (>50%) in the TDP-43 strain relative to the total library. Statistical significance for enrichment within individual compartments was assessed by chi-square test comparing the number of hits and non-hits associated with each compartment (*$p < 0.05$, ***$p < 0.001$). **(H)** Western blot images for PGK1 (loading control), TDP-43, and HSP26 expression in strains harboring TDP-43 and the indicated chaperones.

bioRxiv preprint doi: https://doi.org/10.1101/2025.05.10.653282; this version posted May 10, 2025. The copyright holder for this preprint (which was not certified by peer review) is the author/funder, who has granted bioRxiv a license to display the preprint in perpetuity. It is made available under aCC-BY 4.0 International license.

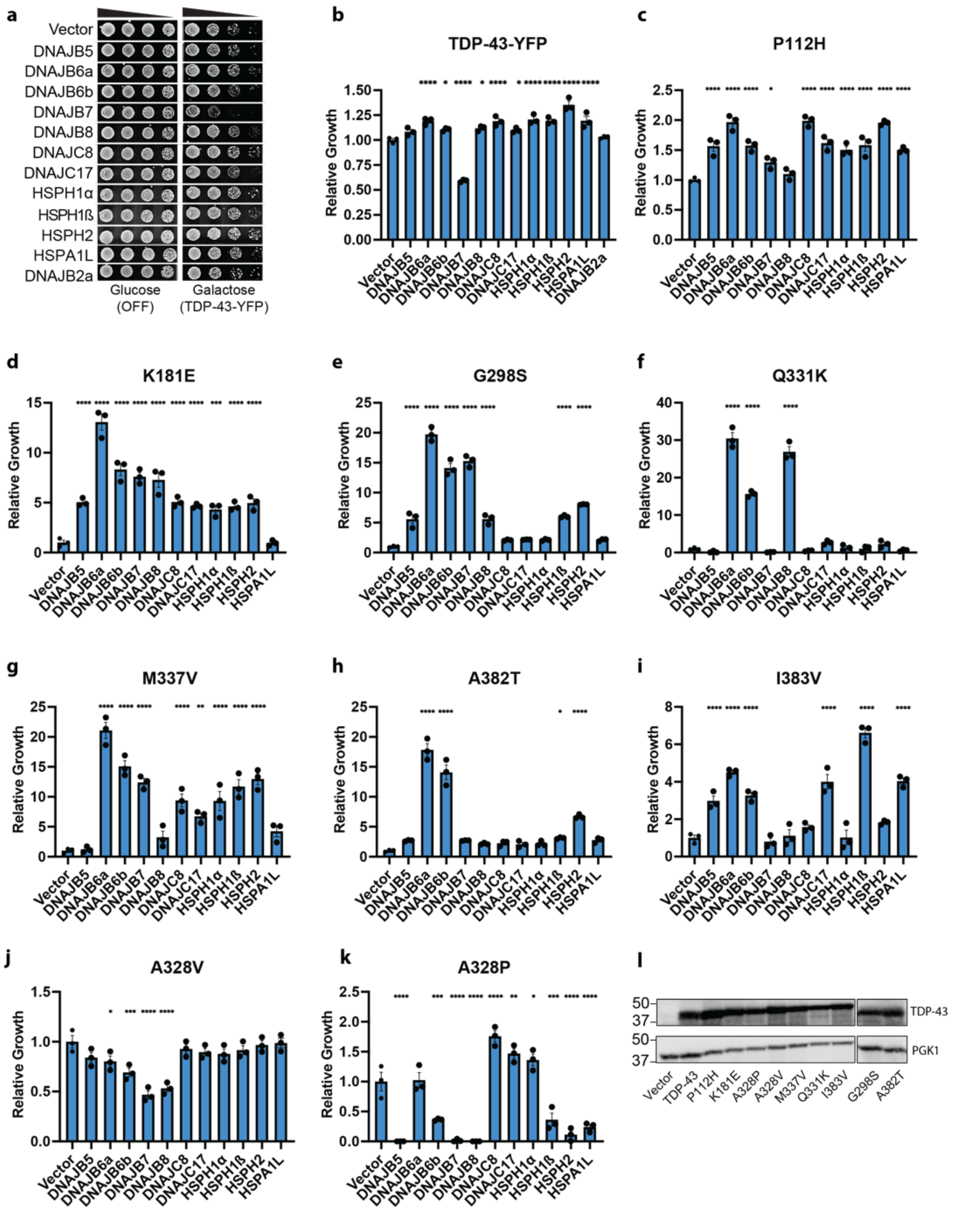

Supplementary Figure 2. Human chaperones buffer the toxicity of diverse synthetic and disease-linked TDP-43 variants. (A) Yeast strains harboring galactose-inducible TDP-43-YFP were transformed with plasmids encoding galactose-inducible human chaperones. On glucose media, there is no expression of TDP-43-YFP or chaperones. Cultures were normalized to equivalent density ($OD_{600} = 2$), serially diluted 5-, 25-, and 125-fold, and spotted onto glucose and galactose agar plates. Images show representative yeast growth. **(B)** Quantification of relative growth normalized to the vector control. Values represent mean ± SEM from three independent replicates. **(C-K)** Quantification of relative growth normalized to the vector control for each indicated TDP-43 variant against the panel of human chaperones. Values represent mean ± SEM from three independent replicates. Statistical significance was calculated relative to the vector control using one-way ANOVA and Dunnett's multiple comparisons test (*$p < 0.05$, **p <

bioRxiv preprint doi: https://doi.org/10.1101/2025.05.10.653282; this version posted May 10, 2025. The copyright holder for this preprint (which was not certified by peer review) is the author/funder, who has granted bioRxiv a license to display the preprint in perpetuity. It is made available under aCC-BY 4.0 International license.

0.01, ***p < 0.001, ****p < 0.0001). **(L)** Western blot images for individual TDP-43 variants confirming their expression. PGK1 is used as a loading control.

bioRxiv preprint doi: https://doi.org/10.1101/2025.05.10.653282; this version posted May 10, 2025. The copyright holder for this preprint (which was not certified by peer review) is the author/funder, who has granted bioRxiv a license to display the preprint in perpetuity. It is made available under aCC-BY 4.0 International license.

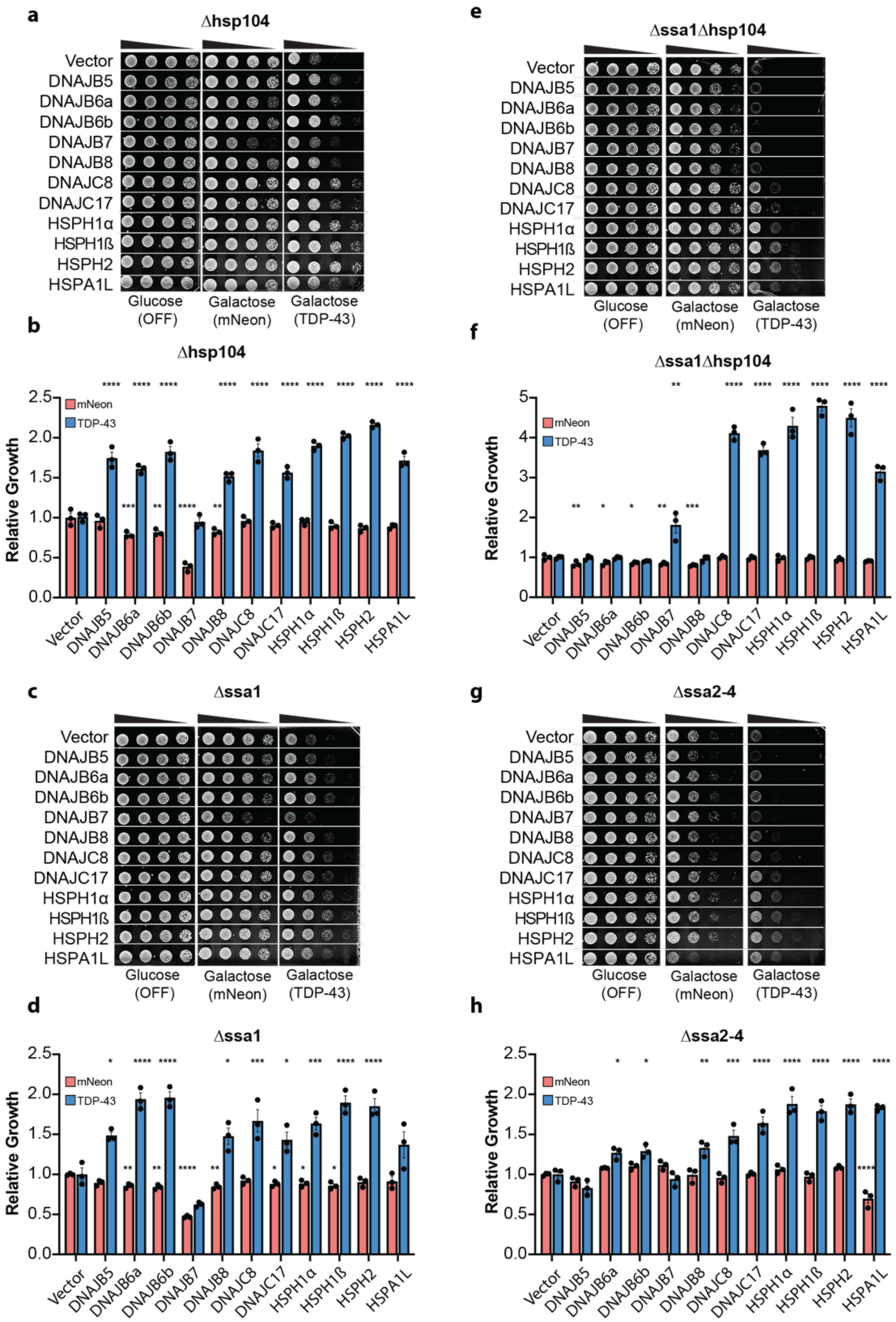

Supplementary Figure 3. DNAJC8, DNAJC17, HSPA1L, HSPH1α, HSPH1β, and HSPH2 suppress TDP-43 toxicity independently of Hsp104 and Ssa1-4. (A) Δhsp104 yeast strains harboring galactose-inducible mNeon or TDP-43 were transformed with plasmids encoding galactose-inducible human chaperones. On glucose media, there is no expression of mNeon, TDP-43, or chaperones. Cultures were normalized to equivalent density (OD_{600} = 2), serially diluted 5-, 25-, and 125-fold, and spotted onto glucose and galactose agar plates. Images show representative yeast growth. **(B)** Quantification of relative growth normalized to the vector control. Values represent mean ± SEM from three independent replicates. **(C-H)** Same as (A, B) for Δssa1 (C, D),

bioRxiv preprint doi: https://doi.org/10.1101/2025.05.10.653282; this version posted May 10, 2025. The copyright holder for this preprint (which was not certified by peer review) is the author/funder, who has granted bioRxiv a license to display the preprint in perpetuity. It is made available under aCC-BY 4.0 International license.

Δ*hsp104*Δ*ssa1* (E, F), Δ*ssa2-4* (G, H). Statistical significance was calculated relative to the vector control using one-way ANOVA and Dunnett's multiple comparisons test (*$p < 0.05$, **$p < 0.01$, ***$p < 0.001$, ****$p < 0.0001$).

bioRxiv preprint doi: https://doi.org/10.1101/2025.05.10.653282; this version posted May 10, 2025. The copyright holder for this preprint (which was not certified by peer review) is the author/funder, who has granted bioRxiv a license to display the preprint in perpetuity. It is made available under aCC-BY 4.0 International license.

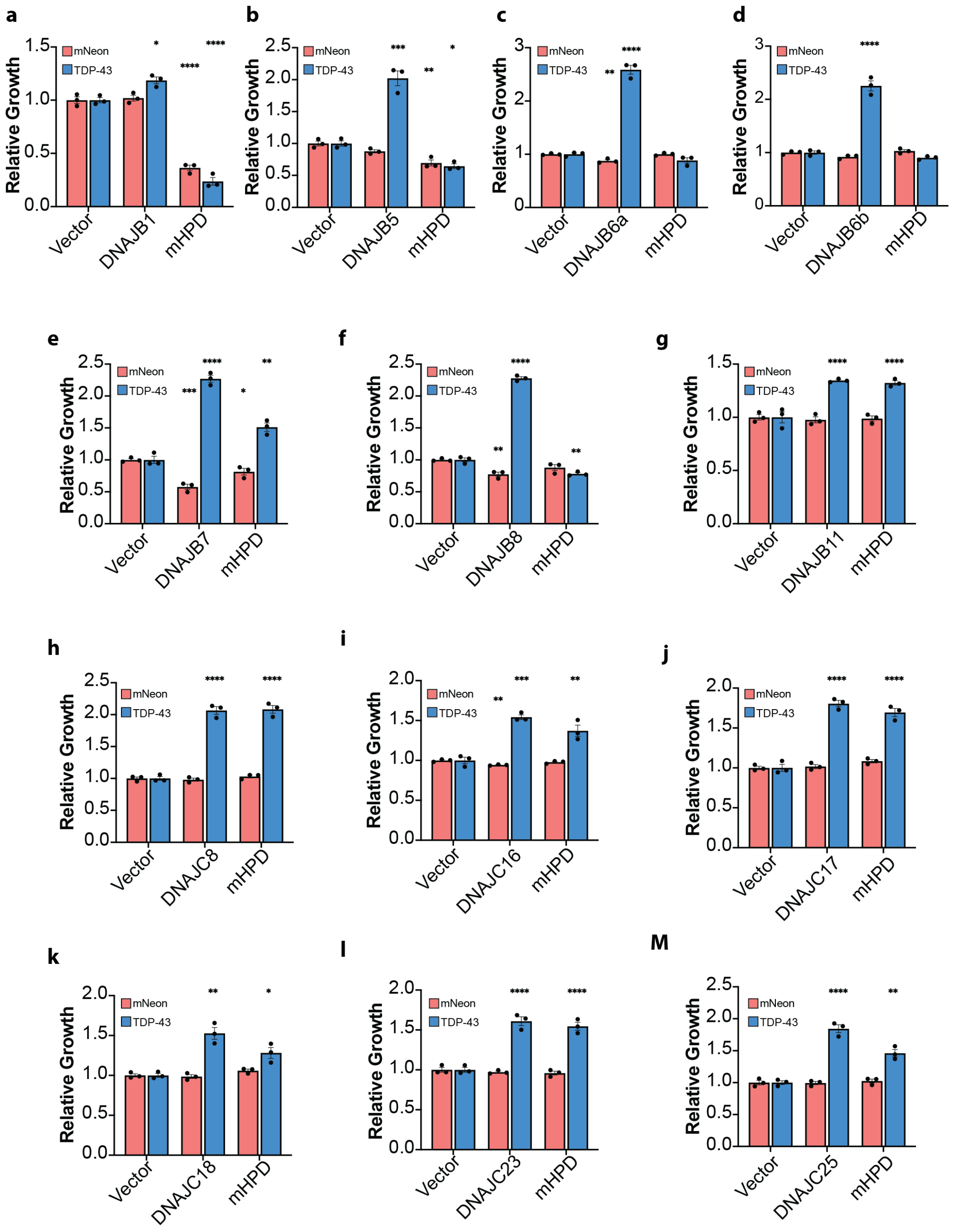

Supplementary Figure 4. HPD motif mutants reveal Hsp70-dependent and Hsp70-independent JDPs. (A-M) Quantification of relative yeast growth for indicated JDP and corresponding mHPD against mNeon or TDP-43. Values represent mean ± SEM of three replicates. Statistical significance was calculated relative to the vector control using one-way ANOVA and Dunnett's multiple comparisons test (*p < 0.05, **p < 0.01, ***p < 0.001, ****p < 0.0001).

bioRxiv preprint doi: https://doi.org/10.1101/2025.05.10.653282; this version posted May 10, 2025. The copyright holder for this preprint (which was not certified by peer review) is the author/funder, who has granted bioRxiv a license to display the preprint in perpetuity. It is made available under aCC-BY 4.0 International license.

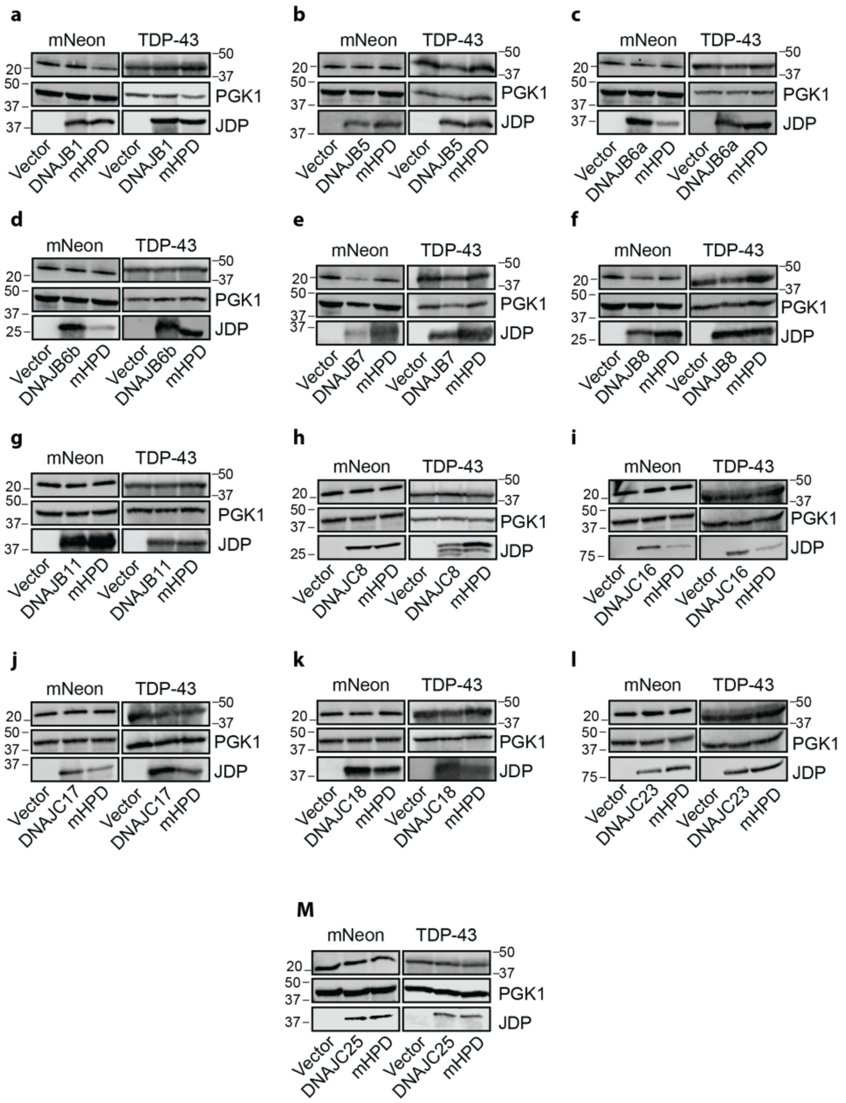

Supplementary Figure 5. Western blots for JDPs and mHPDs. (A-M) TDP-43 and mNeon Western blots for the indicated JDP and corresponding mHPD. PGK1 is used as a loading control. Molecular weight markers are indicated.

bioRxiv preprint doi: https://doi.org/10.1101/2025.05.10.653282; this version posted May 10, 2025. The copyright holder for this preprint (which was not certified by peer review) is the author/funder, who has granted bioRxiv a license to display the preprint in perpetuity. It is made available under aCC-BY 4.0 International license.

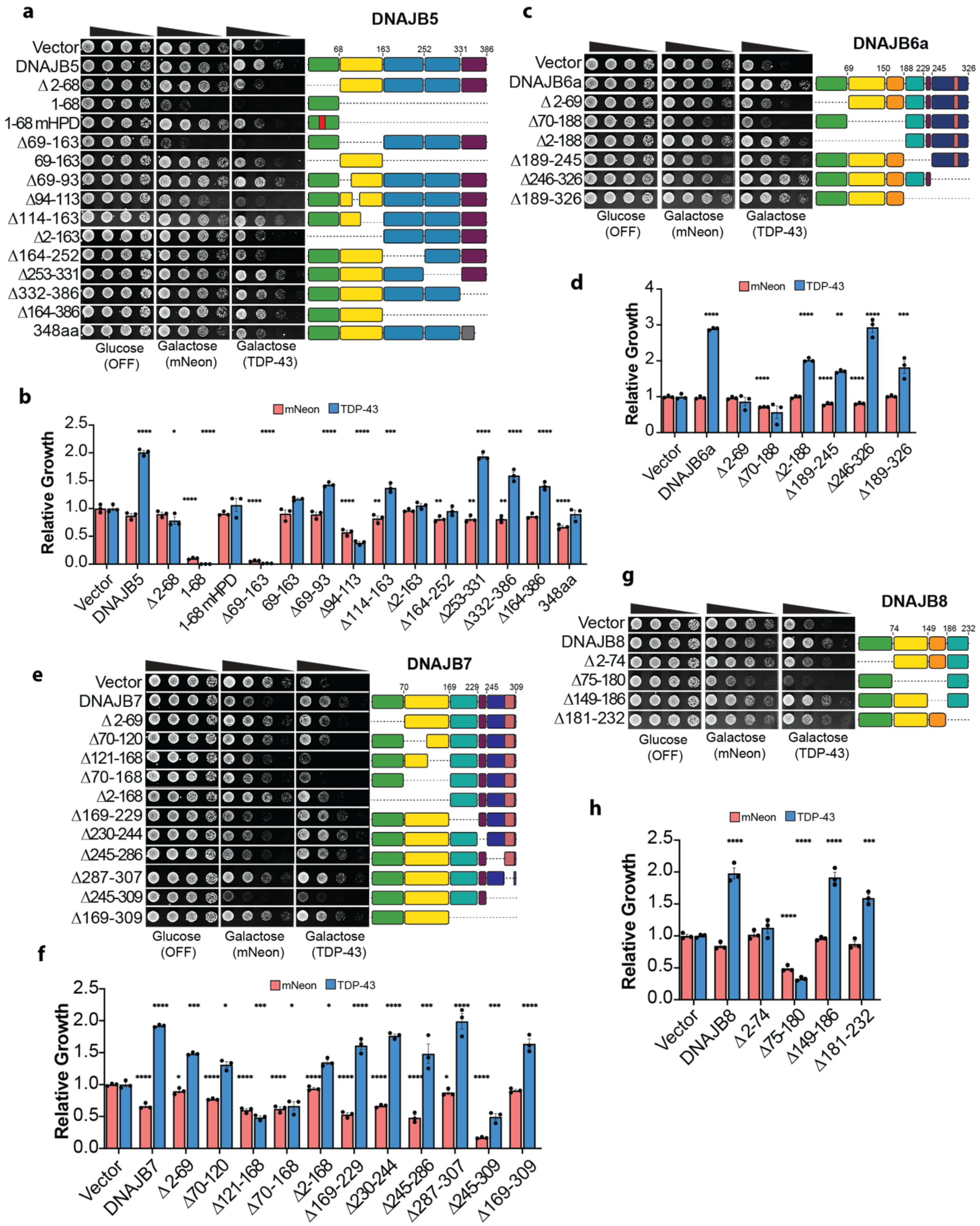

Supplementary Figure 6. Domain deletion analysis for Class B JDPs. (A) Yeast strains harboring mNeon or TDP-43 were transformed with plasmids encoding DNAJB5 domain deletion constructs. Strains were grown on glucose (no mNeon, TDP-43, or chaperone expression) or galactose (induced expression) plates. Cultures were normalized to equivalent density (OD_{600} = 2), serially diluted 5-, 25-, and 125-fold, and spotted onto glucose and galactose plates. Representative yeast growth assay images for DNAJB5 mutants. **(B)** Quantification of

bioRxiv preprint doi: https://doi.org/10.1101/2025.05.10.653282; this version posted May 10, 2025. The copyright holder for this preprint (which was not certified by peer review) is the author/funder, who has granted bioRxiv a license to display the preprint in perpetuity. It is made available under aCC-BY 4.0 International license.

relative yeast growth for DNAJB5 mutants. **(C-H)** Same as **A,B** for DNAJB6a **(C,D)**, DNAJB7 **(E,F)**, and DNAJB8 **(G,H)**. Values are mean ± SEM from three independent replicates. Statistical significance was determined relative to the vector control by one-way ANOVA and Dunnett's multiple comparisons test (*p < 0.05, **p < 0.01, ***p < 0.001, ****p < 0.0001).

bioRxiv preprint doi: https://doi.org/10.1101/2025.05.10.653282; this version posted May 10, 2025. The copyright holder for this preprint (which was not certified by peer review) is the author/funder, who has granted bioRxiv a license to display the preprint in perpetuity. It is made available under aCC-BY 4.0 International license.

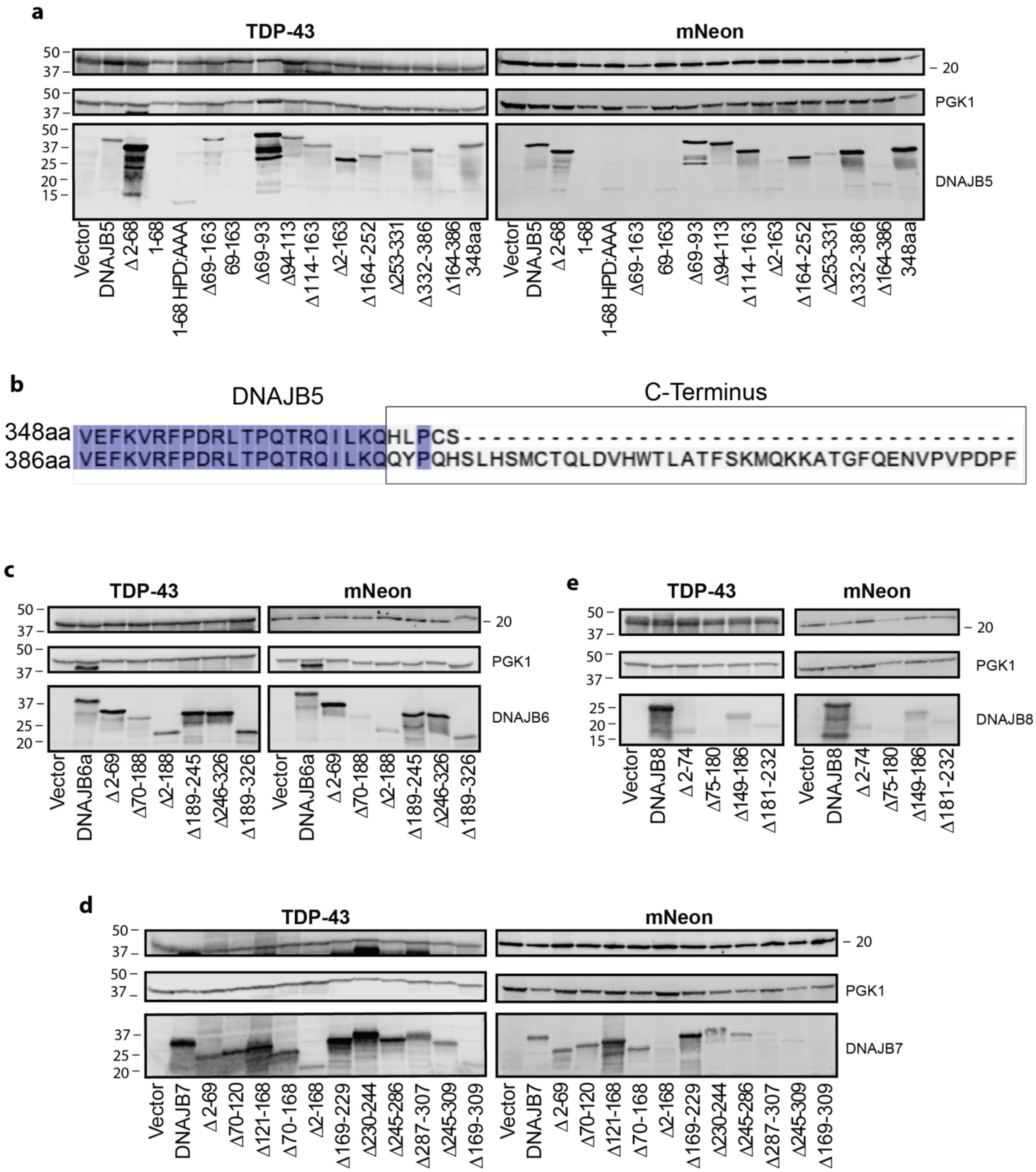

Supplementary Figure 7. Western blots and splice isoform alignment for Class B JDP deletion mutants. (A) TDP-43 and mNeon Western blots for DNAJB5 mutants. PGK1 is used as a loading control. **(B)** Alignment of C-termini for two DNAJB5 splice variants. Colored amino acids are identical between both isoforms. **(C-E)** TDP-43 and mNeon Western blots for DNAJB6a (C), DNAJB7 (D), and DNAJB8 (E) mutants.

bioRxiv preprint doi: https://doi.org/10.1101/2025.05.10.653282; this version posted May 10, 2025. The copyright holder for this preprint (which was not certified by peer review) is the author/funder, who has granted bioRxiv a license to display the preprint in perpetuity. It is made available under aCC-BY 4.0 International license.

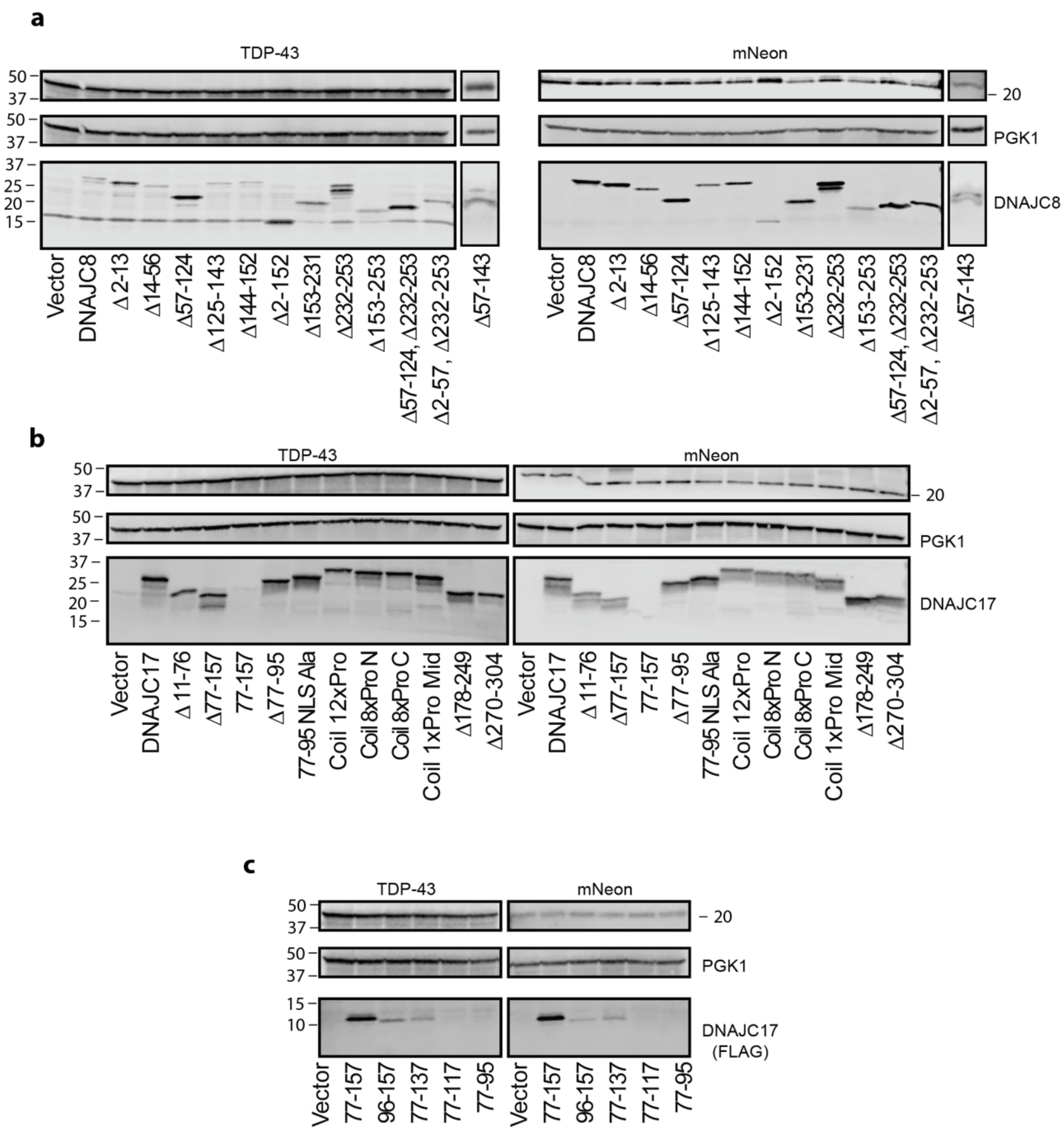

Supplementary Figure 8. Western blots for DNAJC8 and DNAJC17 variants. (A,B) TDP-43 and mNeon Western blots for DNAJC8 (A) and DNAJC17 (B) mutants. **(C)** Western blot detection of FLAG tagged DNAJC17 coiled coil variants. PGK1 is used as a loading control.

bioRxiv preprint doi: https://doi.org/10.1101/2025.05.10.653282; this version posted May 10, 2025. The copyright holder for this preprint (which was not certified by peer review) is the author/funder, who has granted bioRxiv a license to display the preprint in perpetuity. It is made available under aCC-BY 4.0 International license.

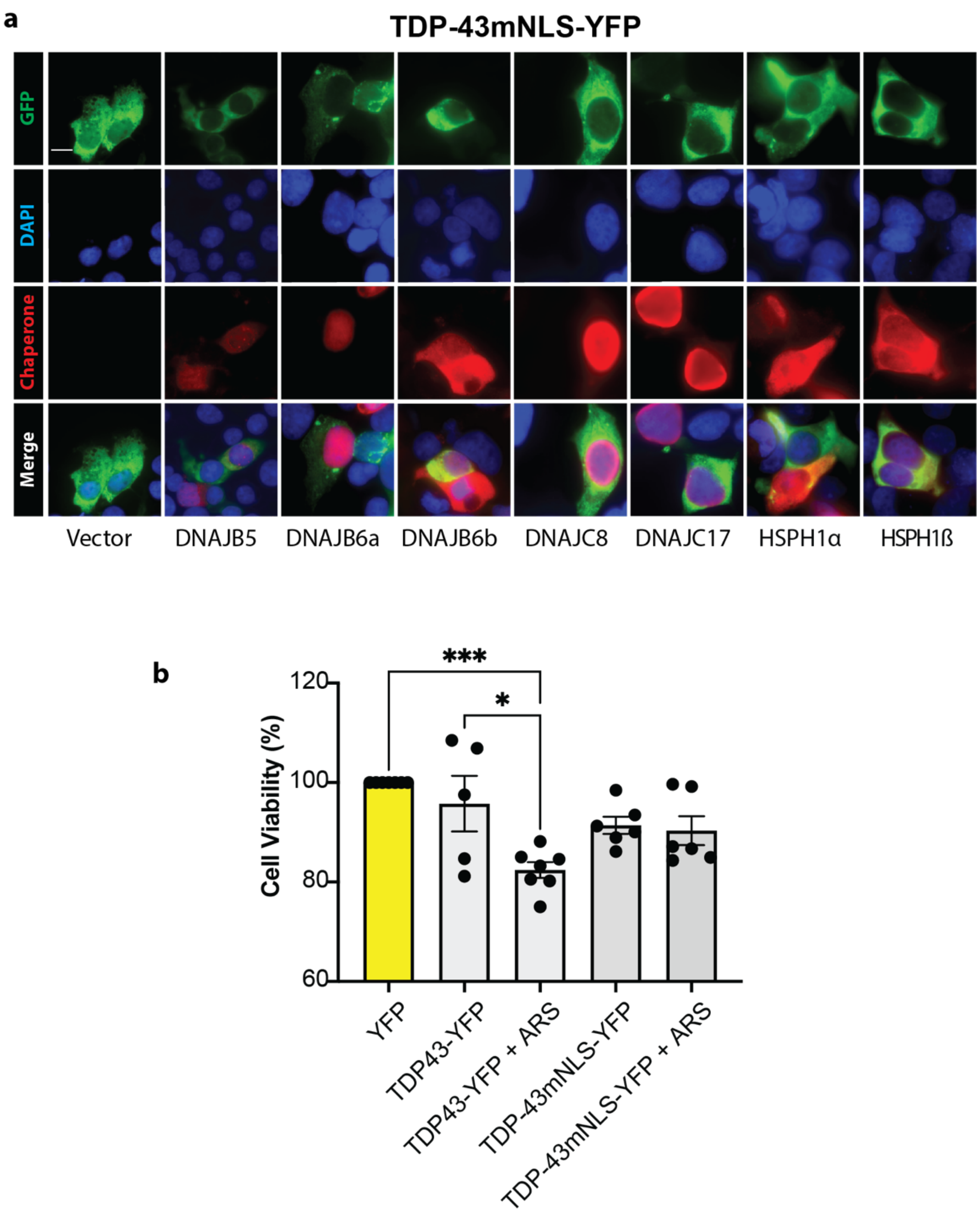

Supplementary Figure 9. Expression of TDP-43-YFP, TDP-43mNLS-YFP, and chaperones in human cells. (A) Representative images showing expression of TDP-43mNLS-YFP and chaperones in HEK293 cells. Cell nuclei are stained with DAPI, and V5-tagged chaperones are detected by immunofluorescence. Scale bar, 20µm. **(B)** Cell viability for cells transfected with YFP, TDP-43-YFP, or TDP-43mNLS-YFP then treated with 5 µM sodium arsenite for 48 hours post transfection. Values are normalized to the YFP control in each experiment and represent mean ± SEM of 5-7 replicates. Statistical significance is determined by one-way ANOVA and Tukey's test (*$p < 0.05$, ***$p < 0.001$).

bioRxiv preprint doi: https://doi.org/10.1101/2025.05.10.653282; this version posted May 10, 2025. The copyright holder for this preprint (which was not certified by peer review) is the author/funder, who has granted bioRxiv a license to display the preprint in perpetuity. It is made available under aCC-BY 4.0 International license.

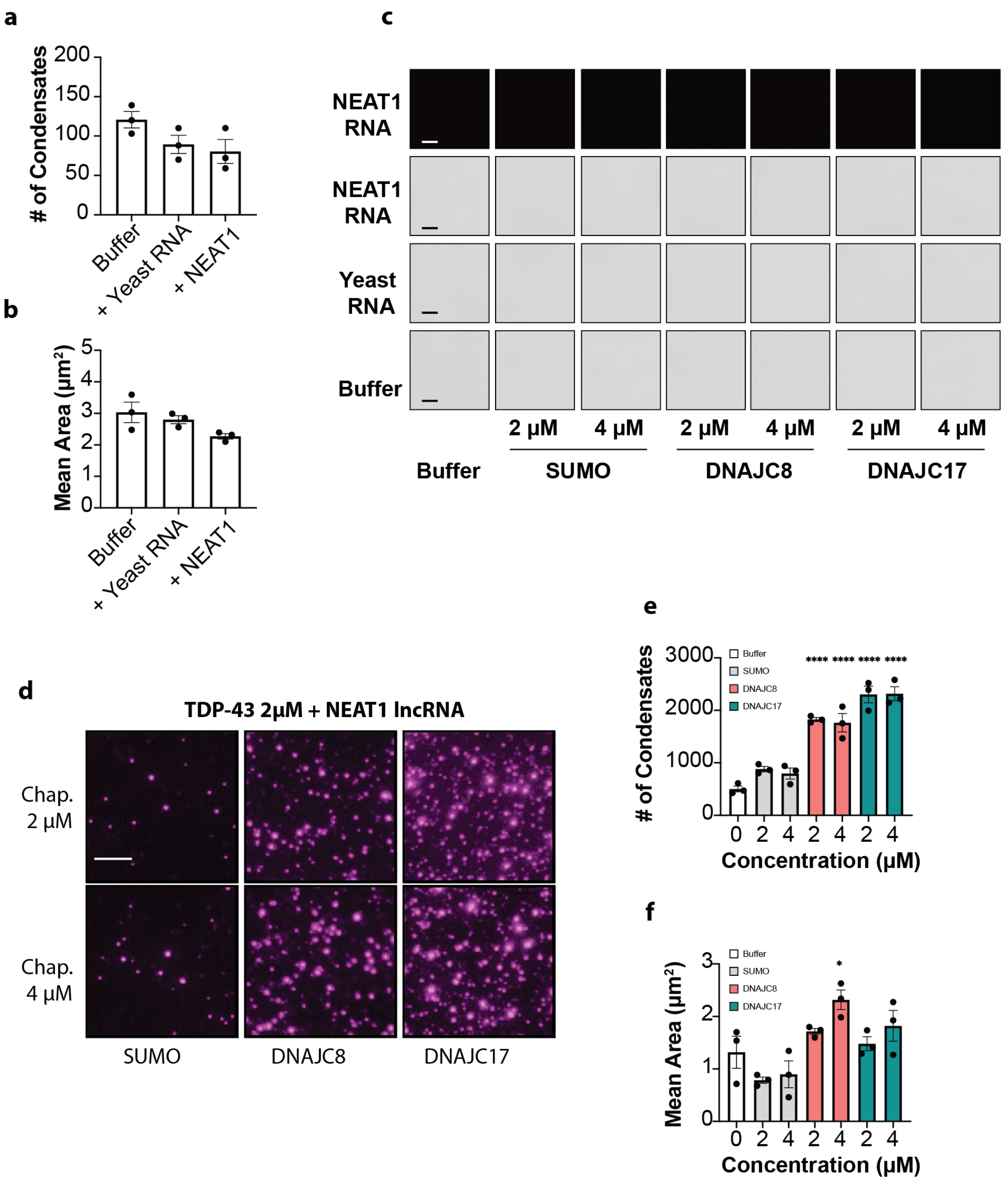

Supplementary Figure 10. Additional controls confirming the enhancement of TDP-43 condensation in the presence of DNAJC8, DNAJC17, and RNA. (A) Quantification of TDP-43 condensate number in buffer with addition of yeast total RNA or NEAT1 lncRNA. Values represent mean ± SEM from three replicates. **(B)** Quantification of TDP-43 condensate area in buffer with addition of yeast total RNA or NEAT1 lncRNA. Values represent mean ± SEM from three replicates. **(C)** Control experiments performed in the absence of TDP-43 showing no detected condensates for the indicated RNAs and chaperones without TDP-43. Scale bar, 5 µm. **(D)**

bioRxiv preprint doi: https://doi.org/10.1101/2025.05.10.653282; this version posted May 10, 2025. The copyright holder for this preprint (which was not certified by peer review) is the author/funder, who has granted bioRxiv a license to display the preprint in perpetuity. It is made available under aCC-BY 4.0 International license.

Representative images showing TDP-43 condensates in the presence of Cy5 labeled NEAT1 lncRNA in buffer with addition of SUMO, DNAJC8, or DNAJC17. Detection of condensates is through the Cy5 fluorescent label confirming the presence of NEAT1 lncRNA in the TDP-43 condensates. Scale bar, 5 µm. **(E, F)** Quantification of TDP-43 condensate number (E) or average area (F) in the presence of Cy5 labeled NEAT1 lncRNA in buffer with addition of SUMO, DNAJC8, or DNAJC17. Values represent mean ± SEM from three replicates. Statistical significance was determined relative to the buffer condition by one-way ANOVA and Dunnett's multiple comparisons test (*$p < 0.05$, ****$p < 0.0001$).
